# Rice cake ileus

**DOI:** 10.1002/ccr3.8587

**Published:** 2024-03-04

**Authors:** Osamu Imataki, Makiko Uemura

**Affiliations:** ^1^ Division of Hematology, Department of Internal Medicine, Faculty of Medicine Kagawa University Kagawa Japan

**Keywords:** glutinous rice, ileus, Japanese rice cake, mochi

## Abstract

Japanese rice cake, mochi, is made from glutinous rice, a very sticky food. Mochi is a popular preserved food during the New Year holiday celebration. However, mochi can be hazardous because of lethal choking or ileus.

## Graphical abstract

Abdominal CT showed melting mochi as a high‐density object in jejunum and ileum.

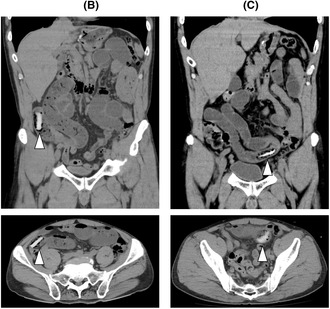

On a cold winter day in December, a 61‐year‐old Japanese man was referred to our emergency clinic after experiencing nausea for 9 h after eating boiled food. His physical examination demonstrated mild tenderness in the middle‐upper abdomen without rebound tenderness. His abdominal X‐ray revealed a stepladder appearance with sentinel air retention (Figure 1A). Computed tomography (CT) scans revealed a 4‐cm‐long flat‐type white matter adjacent to his jejunum (Figure 1B), which indicated undigested Japanese rice cake, as water‐rich rice has a higher density during a CT scan. He recalled that he had swallowed Japanese rice cake at a community assembly. The patient's pertinent medical history included two prior abdominal surgeries secondary to appendicitis and a duodenal ulcer at 25 and 40 years of age, respectively.

**FIGURE 1:**
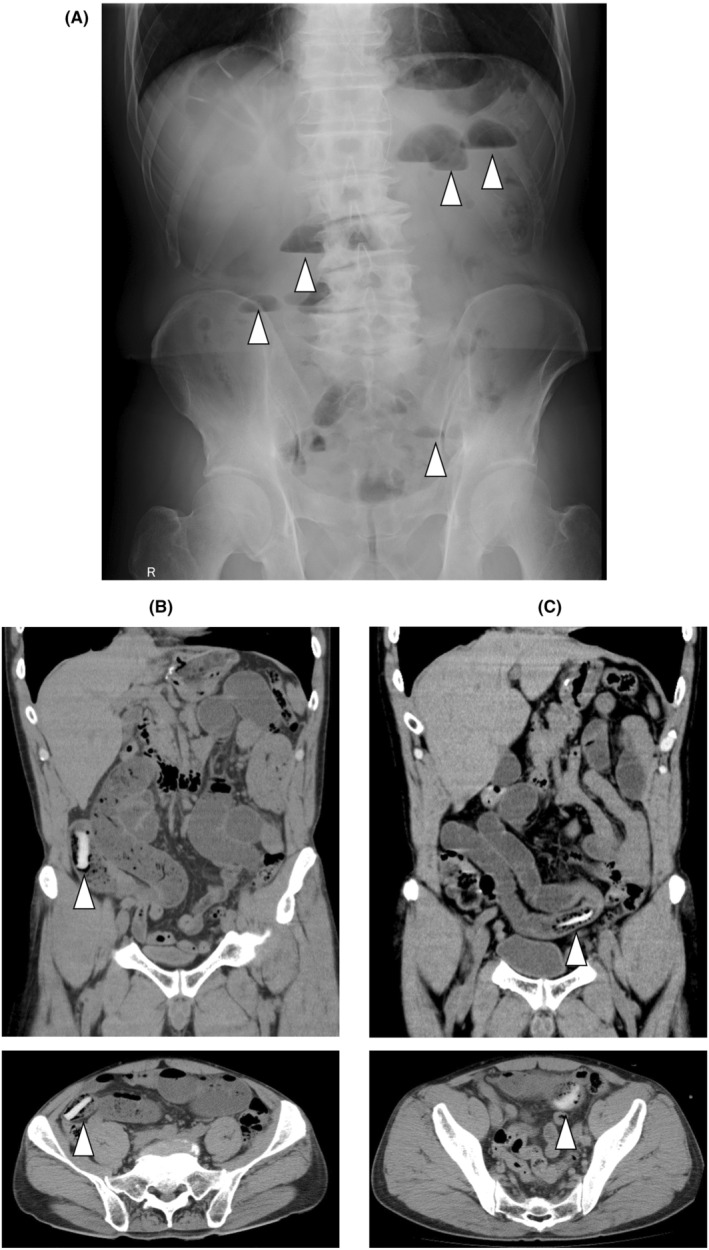
(A) The patient's abdominal X‐ray demonstrated a stepladder appearance with sentinel air retention, which suggested alimentary tract obstruction. (B) The patient's abdominal computed tomography (CT) scans revealed a 4‐cm‐long flat‐type white matter adjacent to his jejunum, which indicated intestinal obstruction due to a high‐density food. (C) A day after admission, the patient's abdominal CT scans showed resolution of intestinal obstruction with a melted high‐density object moved to the anal side.

Japanese rice cake, also called mochi, is made from short‐grain japonica, mochigome. This type of glutinous rice becomes very sticky during processing through steaming, mashing, and pounding. However, mochi is extremely sticky in nature and can induce ileus (rice cake ileus). Or mochi can be a choking hazard in the elderly,^1^, ^2^, ^3^ especially in a new year period.^3^ Mochi is a popular preserved food that is commonly eaten by all Japanese during the New Year holiday season.^3^ Rice cake ileus is another complication that can happen to an individual who enjoys mochi.

Regarding the patient's clinical course, after the insertion of a nasogastric tube, abdominal obstruction resolved on Day 3 of hospitalization. On the day following admission, the primary physician confirmed via CT that the rice cake was digested and was located at the terminal ileum (Figure 1C). Thereafter, enteral feeding was reinitiated. The patient was advised to consume well‐boiled rice cake in a more fluid consistency and not in solid form.

## AUTHOR CONTRIBUTIONS

**Osamu Imataki:** Data curation; formal analysis; funding acquisition; investigation; methodology; project administration; resources; supervision; validation; writing – original draft; writing – review and editing. **Makiko Uemura:** Conceptualization; data curation; funding acquisition; investigation; methodology; project administration; resources; supervision; validation; writing – review and editing.

## FUNDING INFORMATION

This work was supported by funding, JSPS KAKENHI Grant Numbers JP 22K06768 and JP23K11850. We don't have any other financial relationships to disclose.

## CONFLICT OF INTEREST STATEMENT

The authors declare no conflicts of interest.

## ETHICS STATEMENT

We obtained approval from the Kagawa University Hospital Institutional Review Board (H23‐023). This research was conducted ethically in accordance with the World Medical Association Declaration of Helsinki. The subject has given their written informed consent to publish their case (including the publication of images).

## CONSENT

Written informed consent was obtained from the patient for publication of this case series and any accompanying images.

## Data Availability

All data generated or analyzed during this study are included in this published article. Data available on request due to privacy/ethical restrictions.
